# Development of a diagnostic algorithm to ascertain malignant pleural effusion utilizing clinical indicators and serum metal concentrations

**DOI:** 10.3389/fonc.2024.1431318

**Published:** 2024-06-13

**Authors:** Jinling Ji, Ting Shi, Lei Yan, Kai Wang, Kun Jiang, Yuzhang Jiang, Shengnan Pan, Yabin Yu, Chang Li

**Affiliations:** ^1^ Department of Medical laboratory, the Affiliated Huaian No.1 People’s Hospital of Nanjing Medical University, Huaian, Jiangsu, China; ^2^ Department of Hepatobiliary and Pancreatic Surgery, the Affiliated Huaian No.1 People’s Hospital of Nanjing Medical University, Huaian, Jiangsu, China; ^3^ Department of Rheumatology, the Affiliated Huaian No.1 People’s Hospital of Nanjing Medical University, Huaian, Jiangsu, China

**Keywords:** malignant pleural effusion, serum metal, prognosis, indicator, pleural effusion

## Abstract

**Background:**

Malignant pleural effusion (MPE) is prevalent among cancer patients, indicating pleural metastasis and predicting poor prognosis. However, accurately identifying MPE in clinical settings is challenging. The aim of this study was to establish an innovative nomogram-derived model based on clinical indicators and serum metal ion levels to identify MPE.

**Methods:**

From July 2020 to May 2022, 428 patients diagnosed with pleural effusion (PE) were consecutively recruited. Comprehensive demographic details, clinical symptoms, imaging data, pathological information, and laboratory results, including serum metal ion levels, were systematically collected. The nomogram was created by incorporating the most significant predictors identified through LASSO and multivariate logistic regression analysis. The predictors were assigned weighted points based on their respective regression coefficients, allowing for the calculation of a total score that corresponds to the probability of MPE. Internal validation using bootstrapping techniques assessed the nomogram’s performance, including calibration, discrimination, and clinical applicability.

**Results:**

Seven key variables were identified using LASSO regression and multiple regression analysis, including dyspnea, fever, X-ray/CT compatible with malignancy, pleural carcinoembryonic antigen(pCEA), serum neuron-specific enolase(sNSE), serum carcinoembryonic antigen(sCEA), and pleural lactate dehydrogenase(pLDH). Internal validation underscored the superior performance of our model (AUC=0.940). Decision curve analysis (DCA) analysis demonstrated substantial net benefit across a probability threshold range > 1%. Additionally, serum calcium and copper levels were significantly higher, while serum zinc levels were significantly lower in MPE patients compared to benign pleural effusion (BPE) patients.

**Conclusion:**

This study effectively developed a user-friendly and reliable MPE identification model incorporating seven markers, aiding in the classification of PE subtypes in clinical settings. Furthermore, our study highlights the clinical value of serum metal ions in distinguishing malignant pleural effusion from BPE. This significant advancement provides essential tools for physicians to accurately diagnose and treat patients with MPE.

## 1 Introduction

PE, a common clinical condition, is classified as benign or malignant based on its underlying etiology. PE can result from various underlying conditions, such as congestive heart failure, renal insufficiency, severe pneumonia, or hypoproteinaemia, which disrupt the peritoneal or protein equilibrium, leading to pathological fluid accumulation in the pleural cavity, complicating both diagnosis and therapeutic interventions. MPE, associated with neoplastic disorders, accounts for approximately 10% of all PE cases and is linked to diverse tumor types such as lymphoma, sarcoma, lung, breast, ovarian, gastric, and colorectal carcinomas. The presence of MPE introduces considerable clinical complexities, emphasizing the critical importance of accurately distinguishing between BPE and MPE (1, 2).This precise differentiation is not only vital for achieving an accurate diagnosis but is also paramount in designing effective treatment strategies tailored to the specific nature of the PE (3, 4). Accurate differentiation enables clinicians to design targeted therapeutic approaches, thereby optimizing patient care and outcomes in the management of PE (5, 6).

Accurate diagnosis of BPE and MPE requires a comprehensive assessment of pleural fluid, incorporating cytological analysis, thoracoscopic evaluations, and biochemical markers, including tumor-specific indicators (7, 8). Alongside pleural fluid examination, symptom evaluation plays a crucial role in this diagnostic process. Although cytological analysis remains standard for detecting MPE, its sensitivity was only 63% in a study of 725 MPE patients (9). This method’s subjective nature, heavily reliant on the pathologist’s expertise, introduces variability in diagnosis. While pleural biopsy offers higher detection rates for MPE, its invasive nature can complicate patient consent and lead to diagnostic delays, impacting disease management and outcomes (10). Recent findings by Wang et al. (11) identified S100A2 as a potential biomarker for distinguishing between MPE and tuberculous PE (TPE), although the assay’s availability remains limited in resource-constrained settings.

Serum metal ion levels, including iron, copper, and zinc, show significant variations in oncological and non-oncological conditions. Elevated copper and iron levels are often observed in cancers such as hepatocellular carcinoma and breast cancer, reflecting their roles in cellular proliferation and angiogenesis. Conversely, in non-neoplastic conditions like inflammatory diseases or ascites, metal ion concentrations may decrease or vary, reflecting malabsorption or metabolic disturbances caused by the underlying disease. These alterations suggest potential as biomarkers for diagnosis and therapeutic monitoring. However, the specific alterations of serum metal ions in patients with MPE versus BPE remain underexplored.

This study aimed to develop a diagnostic model that effectively differentiates MPE from other conditions by evaluating the predictive value of readily available demographic characteristics and laboratory markers in patients with PE.

## 2 Materials and methods

### 2.1 Patients and ethical approval

**Figure 1:**
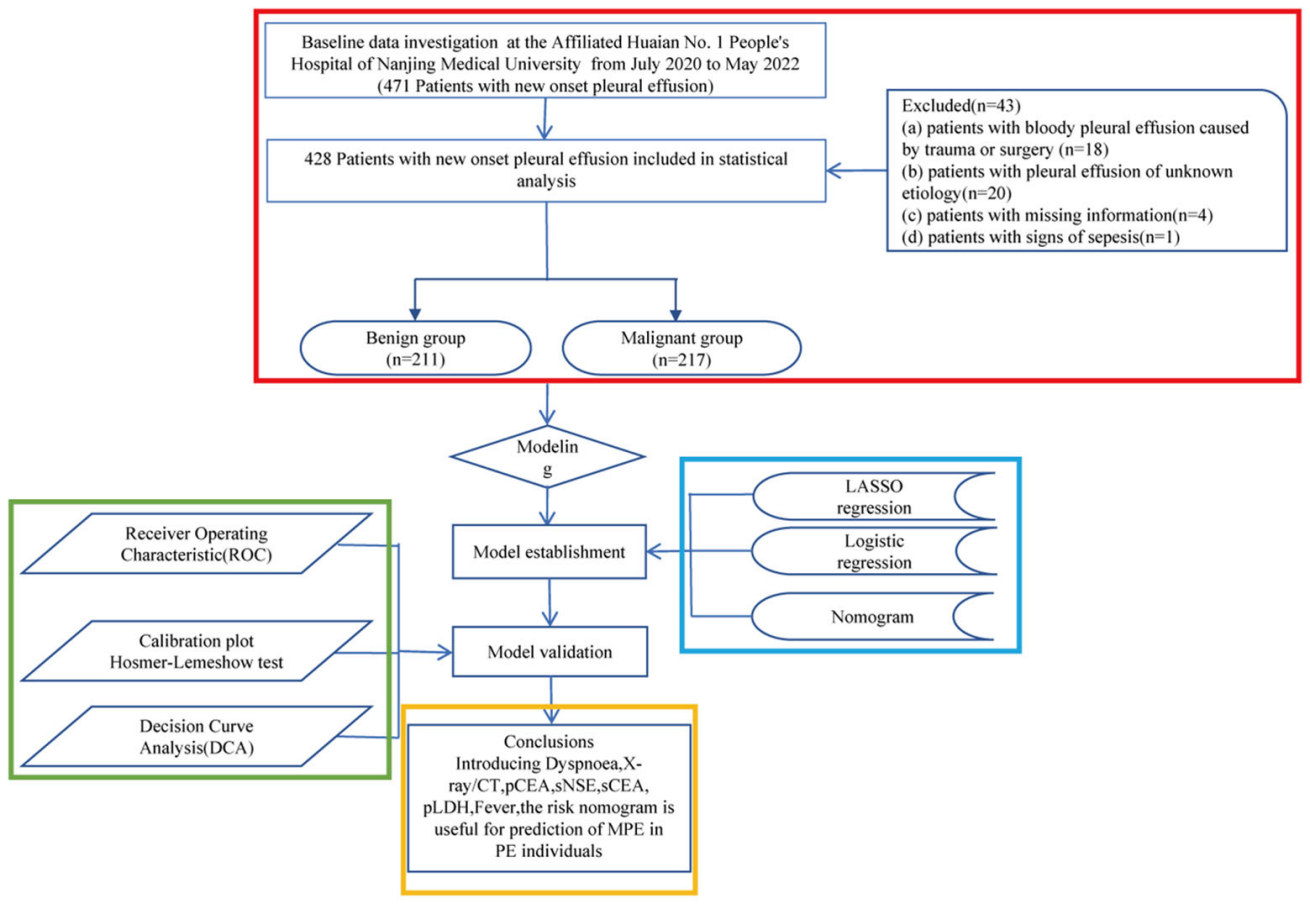
Flowchart illustrating patient inclusion.

This retrospective cohort study included patients diagnosed with PE at the Affiliated Huaian No. 1 People’s Hospital of Nanjing Medical University between July 2020 and May 2022. The inclusion criteria were: (a) recent PE diagnosis, (b) undergoing diagnostic thoracentesis, and (c) informed agreement to participate. Exclusion criteria were: (a) trauma- or surgery-related hemorrhagic PE, (b) PE of unknown origin, (c) incomplete patient data, and (d) signs of sepsis. Originally 471 patients were considered, with 43 subsequently excluded based on these criteria. The patient selection process is depicted in **Figure 1**.

Ethical approval was granted by the Ethics Committee of Huaian No.1 People’s Hospital of Nanjing Medical University (KY-2023–023-01). The retrospective and anonymized nature of the study waived the need for signed informed consent, a common practice in studies where data are de-identified.

### 2.2 Data collection

Before the collection of pleural fluid samples and clinical data used in the development of the predictive model, all patients were subjected to a standardized protocol. This protocol was implemented prior to initiating any interventions, such as the administration of intravenous fluids, diuretics, chemotherapy, acupuncture, or other therapeutic approaches that could potentially influence the pleural fluid composition or clinical presentation. By ensuring that samples and data were obtained before treatment initiation, we aimed to minimize the confounding effects of therapeutic interventions on the model’s predictive accuracy. These included thoracentesis and blood sample collection to establish baseline diagnostics and assess patient condition prior to treatment initiation. Demographic and clinical data were meticulously gathered from patient records, including age, sex, smoking history, symptoms (dyspnoea, chest pain), general syndrome, fever, imaging (X-ray/CT) indicative of malignancy, and comorbid conditions (hypertension, diabetes). Laboratory parameters measured included platelet counts, haemoglobin, albumin, sCEA, pCEA, sSCCA, sNSE, pLDH, and total serum protein levels.

### 2.3 Diagnostic criteria

Three independent researchers reviewed patient data to determine the etiology of PE. Inconsistent cases were excluded. A total of 428 patients were classified into BPE and MPE groups based on established diagnostic criteria. Diagnostic criteria for MPE included: (1) positive cytology in PE fluid, (2) positive diagnostic pleural biopsy, and (3) evidence of a primary tumor or metastasis, excluding other potential causes.

### 2.4 Nomogram construction and validation

Baseline characteristics were summarized using descriptive statistics. Group comparisons were performed using Pearson’s chi-square or Fisher’s exact tests as appropriate. Based on expert knowledge and prior information, covariates associated with the missing variable should be carefully selected. These covariates include other completely observed variables and the observed values of the missing variable itself. In the logistic regression model, the missing variable is treated as the dependent variable, while the selected covariates serve as independent variables. The model is then used to impute the missing values. Predictor selection and regularization were conducted using the LASSO regression method, followed by multivariate logistic regression to construct a prognostic nomogram. The model’s discriminative ability was evaluated by calculating the area under the curve (AUC). Internal validation was performed using bootstrapping (500 iterations) to ensure robustness. Model calibration was assessed via the Hosmer-Lemeshow test (12). The clinical utility of the nomogram was further evaluated using DCA (13).

### 2.5 Serum metal level assay

Whole blood samples were collected into K2 EDTA BD Vacutainer tubes and processed within one hour. Samples were centrifuged at 820 g for 10 minutes at room temperature to separate plasma, which was further centrifuged at 16,000 g for 10 minutes to precipitate cellular debris. The supernatant was stored at -80°C. Concentrations of serum calcium, magnesium, copper, and zinc were determined using enzyme-linked immunoassay kits (Solarbio for calcium BC0720 and magnesium BC2795, and Qiyibio for zinc QYS-239485, China).

### 2.6 Statistical analysis

Statistical analyses were conducted using R software (v3.6.3), with a p-value of <0.05 indicating statistical significance. Laboratory data were presented as mean ± SD. Data visualization was carried out using GraphPad Prism 8.3 (GraphPad Software, San Diego, CA).

## 3 Results

### 3.1 Patient features

This investigation enrolled 471 patients newly diagnosed with PE. A subset of 43 patients was excluded from subsequent analyses due to deviations from the specified inclusion criteria (**Figure 1**). Thus, 428 patients were analyzed in detail, with their etiological classifications presented in **Table 1**. Patients were categorized into benign and malignant groups; among the benign group (BPE, n=211), pneumonia was the most prevalent etiology, accounting for 39.3% of cases, followed by chronic obstructive pulmonary disease (COPD) at 11.8%, and empyema at 8.1%. In the malignant group (MPE, n=217), lung adenocarcinoma was the primary diagnosis in 63.6% (138/217) of cases, with squamous cell carcinoma following at 7.8% (17/217). Pathological cell examination yielded positive results in 103 of the 217 MPE cases, whereas all 94 cases in the benign cohort tested negative, culminating in a sensitivity for MPE diagnosis of 47.5% (103/217) and a specificity of 100% (211/211), thereby achieving a diagnostic accuracy of 73.4% (314/428).

**Table 1:** The aetiology of pleural effusion among the patients included in this research.

Aetiology	Benign group (n=211)	Malignant group (n=217)	Total
			(n=428)
Pneumonia	83	0	83
COPD	25	0	25
Empyema	17	0	17
Tuberculous pleurisy	10	0	10
Miscellaneous	76	0	76
Adenocarcinoma of the lung	0	138	138
Squamous cell carcinoma of the lung	0	17	17
Esophageal cancer	0	15	15
Small cell carcinoma of the lung	0	11	11
Breast cancer	0	5	5
Other cancers	0	31	31

COPD, chronic obstructive pulmonary disease.

### 3.2 Baseline characteristics and risk factors for MPE

The study comprised 428 participants, 50.7% (217/428) of whom were diagnosed with MPE. Detailed demographic and clinical characteristics are delineated in **Table 2**. Initial assessments highlighted a higher incidence of MPE in female, accompanied by symptoms such as dyspnea, chest pain, and systemic symptoms, and radiologic findings suggestive of malignancy. Notably elevated levels of sCEA, sNSE, pCEA, pLDH, hypoalbuminemia, and anemia were observed, whereas fever was less common (P < 0.05).

**Table 2:** Features of the participants.

Variables	Total, N (%)	PE		*p*	statistic
		Benign group, N (%)	Malignant group, N (%)		
Total patients	428	211	217		
Gender				0.004	χ2 = 8.504
Female	152 (36)	60 (28)	92 (42)		
Male	276 (64)	151 (72)	125 (58)		
Age,year				0.258	χ2 = 1.278
<60	142 (33)	64 (30)	78 (36)		
≥60	286 (67)	147 (70)	139 (64)		
Smoking history				0.857	χ2 = 0.032
No	309 (72)	151 (72)	158 (73)		
Yes	119 (28)	60 (28)	59 (27)		
Hypertension				0.128	χ2 = 2.311
No	325 (76)	153 (73)	172 (79)		
Yes	103 (24)	58 (27)	45 (21)		
Diabetes				0.669	χ2 = 0.183
No	389 (91)	190 (90)	199 (92)		
Yes	39 (9)	21 (10)	18 (8)		
Dyspnoea				< 0.001	χ2 = 43.073
No	154 (36)	109 (52)	45 (21)		
Yes	274 (64)	102 (48)	172 (79)		
Chest pain				0.031	χ2 = 4.644
No	286 (67)	130 (62)	156 (72)		
Yes	142 (33)	81 (38)	61 (28)		
General syndrome				< 0.001	χ2 = 13.237
No	155 (36)	95 (45)	60 (28)		
Yes	273 (64)	116 (55)	157 (72)		
Fever				< 0.001	χ2 = 59.079
No	264 (62)	91 (43)	173 (80)		
Yes	164 (38)	120 (57)	44 (20)		
X-ray/CT				< 0.001	χ2 = 137.186
No	197 (46)	158 (75)	39 (18)		
Yes	231 (54)	53 (25)	178 (82)		
Anemia				< 0.001	χ2 = 19.835
No	218 (51)	131 (62)	87 (40)		
Yes	210 (49)	80 (38)	130 (60)		
Hypoproteinemia				< 0.001	χ2 = 12.013
No	334 (78)	180 (85)	154 (71)		
Yes	94 (22)	31 (15)	63 (29)		
sCEA,ug/L				< 0.001	χ2 = 122.466
≤5	268 (63)	188 (89)	80 (37)		
>5	160 (37)	23 (11)	137 (63)		
sNSE,ug/L				< 0.001	χ2 = 26.106
≤16.3	242 (57)	146 (69)	96 (44)		
>16.3	186 (43)	65 (31)	121 (56)		
sSCCA,ug/L				0.461	χ2 = 0.544
≤2.7	388 (91)	194 (92)	194 (89)		
>2.7	40 (9)	17 (8)	23 (11)		
pCEA,ug/L				< 0.001	χ2 = 152.112
≤30	282 (66)	200 (95)	82 (38)		
>30	146 (34)	11 (5)	135 (62)		
pLDH,ug/L				< 0.001	χ2 = 27.530
≤246	178 (42)	115 (55)	63 (29)		
>246	250 (58)	96 (45)	154 (71)		
pTP,g/L				1	Fisher
63∼82	9 (2)	4 (2)	5 (2)		
≤63 or >82	419 (98)	207 (98)	212 (98)		

PE, pleural effusion; sCEA, serum carcinoembryonic antigen; sNSE, serum neuron-specific enolase; sSCC, serum squamous cell carcinoma antigen; pCEA, pleural carcinoembryonic antigen; pLDH, pleural lactate dehydrogenase; pTP, pleural effusion total protein.

### 3.3 Model development and validation

**Figure 2:**
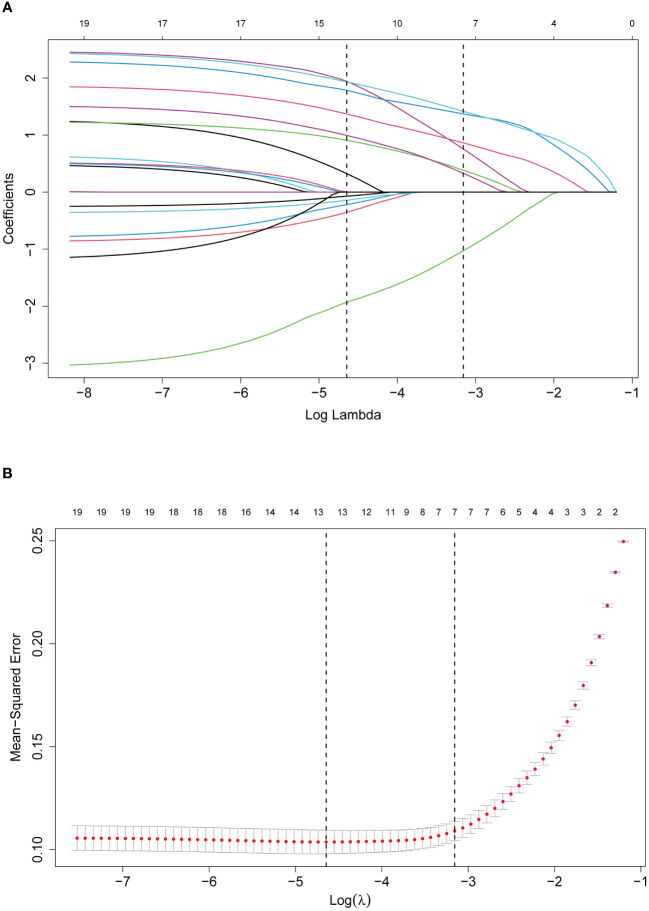
Selection of predictors through the LASSO regression analysis. **(A)** The process involved tuning parameter (lambda) selection via 10-fold cross-validation and plotting binomial deviance against log (lambda). The dotted vertical lines were plotted at the optimal values as per the 1-SE criteria. **(B)** LASSO regression coefficient profiles of variables. A coefficient profile plot was generated against the log (lambda) sequence. Seven non-zero coefficients were selected and employed to establish the prognostic model.

**Figure 3:**
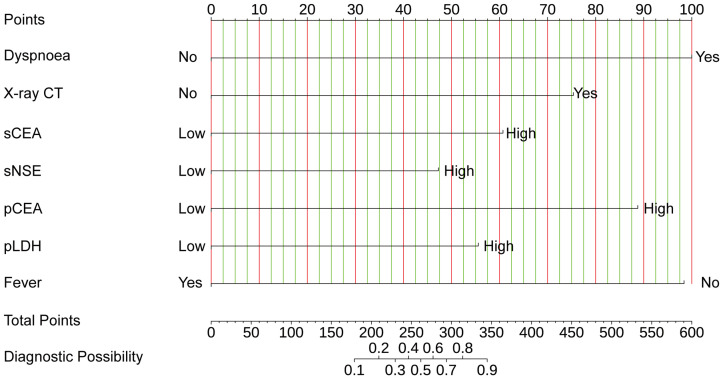
The nomogram offers a means to predict the risk of MPE. When using the nomogram, the points for each predictor (variable) of a patient on the uppermost rule should be located. Subsequently, the total points are obtained by adding all points. Ultimately, the corresponding predicted probability of MPE on the lowest rule is identified.

LASSO regression analysis identified seven significant predictors of MPE (**Figure 2**): dyspnea, fever, X-ray/CT findings compatible with malignancy, sCEA, sNSE, pCEA, and pLDH. These predictors were incorporated into a multivariable logistic regression model to construct a nomogram for predicting MPE probability, with coefficients calculated as follows: dyspnea (2.650), fever (-2.607), X-ray/CT (1.997), pCEA (2.352), sCEA (1.609), sNSE (1.253), and pLDH (1.473) (**Figure 3**). The resultant logistic regression formula is logistic (risk score) = -4.327 + 2.650 × dyspnea - 2.607 × fever + 1.997 × X-ray/CT + 1.609 × sCEA + 2.352 × pCEA + 1.473 × pLDH + 1.253 × sNSE (**Table 3**).

**Table 3:** Estimated ORs obtained in a logistic regression model (backward Wald).

Characteristics	B	SE	OR	95%CI	Z	*P*
Dyspnoea	2.650	0.497	14.148	5.570∼39.460	5.335	<0.001
X-ray/CT	1.997	0.367	7.365	3.651∼15.52	5.435	<0.001
sCEA	1.609	0.414	4.997	2.250∼11.48	3.89	<0.001
sNSE	1.253	0.352	3.500	1.780∼7.122	3.559	<0.001
pCEA	2.352	0.479	10.507	4.249∼28.17	4.906	<0.001
pLDH	1.473	0.376	4.361	2.128∼9.372	3.913	<0.001
Fever	-2.61	0.432	0.074	0.030∼0.165	-6.04	<0.001

OR, odds ratio; CI, confidence interval; X-ray/CT, images in chest X-ray/CT suggestive of malignancy; sCEA, serum carcinoembryonic antigen; sNSE, serum neuron-specific enolase; pCEA, pleural carcinoembryonic antigen; pLDH, pleural lactate dehydrogenase.

**Figure 4:**
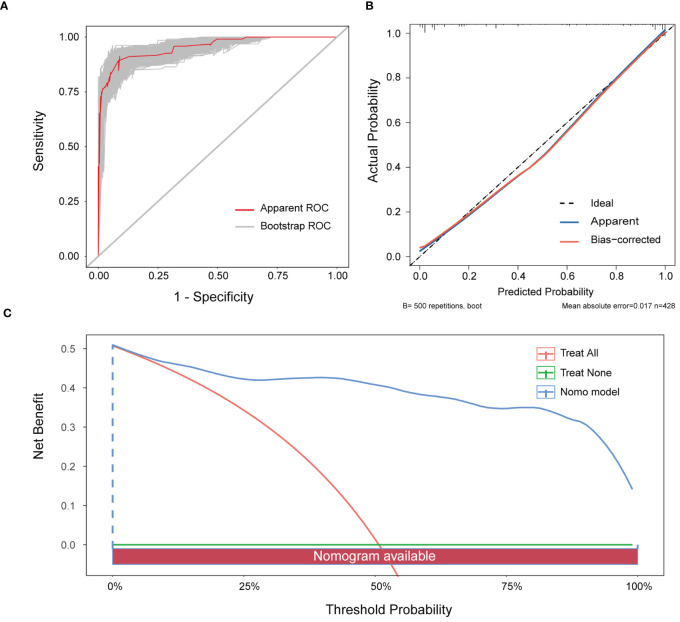
Verification of the nomogram model. **(A)** ROC curve for the nomogram generated utilising bootstrap resampling. **(B)** Nomogram calibration plot. The proximity of the solid line (signifying performance nomogram) to the dotted line (representing the ideal model) indicates the accuracy of the predictions of the nomogram. **(C)** Decision curve analysis of the nomogram. The red solid line represents the nomogram. The graph displays the expected net benefit per patient in relation to the MPE risk predicted by the nomogram. The solid horizontal line represents patients without MPE, while the grey line represents those with MPE.

The nomogram’s performance was evaluated using multidimensional validation methods. The area under the curve (AUC) was 0.940 (95% confidence interval: 0.919–0.962), as determined by bootstrap resampling (n=500), demonstrating robust discriminative capacity (**Figure 4A**). The calibration plot (**Figure 4B**) showed excellent agreement between predicted probabilities and observed outcomes. Furthermore, the Hosmer-Lemeshow test yielded a P-value of 0.601, indicating no significant deviation between expected and observed outcomes, thereby confirming the model’s fit and reliability. DCA revealed that the nomogram provided greater net benefit than either ‘screen-none’ or ‘screen-all’ strategies across a threshold probability >1% (**Figure 4C**), emphasizing its utility in clinical decision-making for the screening and management of MPE.

### 3.4 Models comparison

**Figure 5:**
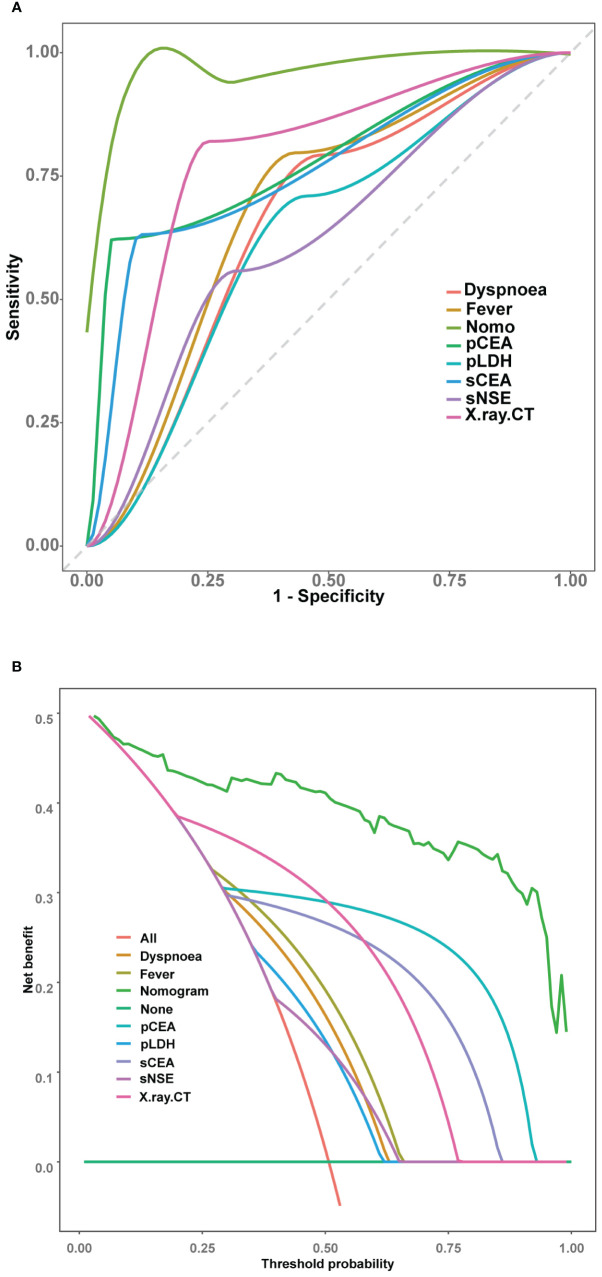
Comparison of the models in the entire study cohort. **(A)** Receiver operating characteristic curves of various models. **(B)** Decision curve analysis of various models.

The receiver operating characteristic (ROC) curve analyses (**Figure 5A**) and DeLong tests were employed to compare the discriminative efficacy of the nomogram (AUC = 0.940) against individual variables. The nomogram demonstrated superior performance compared to dyspnoea (AUC = 0.655, P < 0.001), fever (AUC = 0.685, P < 0.001), X-ray/CT (AUC = 0.785, P < 0.001), sCEA (AUC = 0.761, P < 0.001), pCEA (AUC = 0.785, P < 0.001), pLDH (AUC = 0.627, P < 0.001), and sNSE (AUC = 0.625, P < 0.001).

Further evaluation and comparison of the clinical utility of the models were performed using DCA. As depicted in **Figure 5B**, the nomogram consistently outperformed the models that utilized only the individual risk factors, providing superior overall net benefit across a wide range of threshold probabilities.

### 3.5 Serum levels of metal ions differentiate MPE from BPE

**Figure 6:**
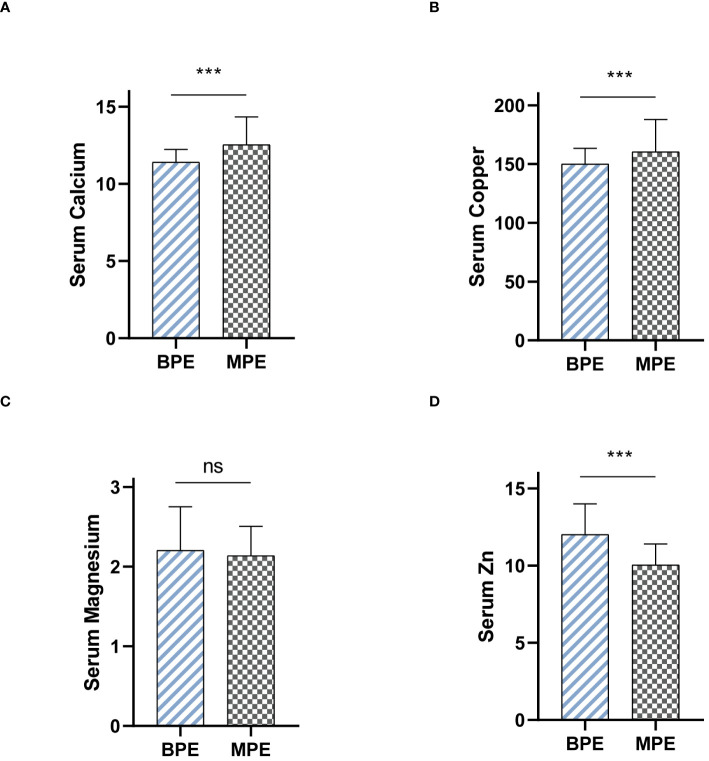
Serum levels of metal ions differentiate MPE from BPE. **(A)** Serum Calcium levels between BPE and MPE. **(B)** Serum Copper levels between BPE and MPE. **(C)** Serum Magnesium levels between BPE and MPE. **(D)** Serum Zinc levels between BPE and MPE. ns, not significant. ***P < 0.001.

We collected serum samples from patients presenting with MPE and BPE, and subsequently measured differences in the levels of calcium, copper, magnesium, and zinc. Notably, in patients diagnosed with MPE, the levels of serum calcium and copper were significantly higher than those in patients with BPE (**Figures 6A, B**). Conversely, serum zinc demonstrated an opposite trend (**Figure 6D**). Additionally, no significant differences in serum magnesium levels were observed between the two patient groups (**Figure 6C**).

## 4 Discussion

Historically, the differentiation of PE types has relied on specific diagnostic techniques like pleural biopsy, diagnostic thoracentesis, and PE cytology (14). However, the sensitivity of cytology in this context, reported at 47.5%, did not meet satisfactory diagnostic standards, prompting further research into MPE-related factors including diverse immunophenotypes (15). A noteworthy study involved single-cell RNA sequencing of 62,382 cells from patients with non-small-cell lung cancer-associated MPE, aiming to delineate the immune cell landscape within MPE (16). This study provided valuable insights into the interactions among different T-helper cell subtypes and their roles in the tumor and mesothelial environments. Despite their potential diagnostic significance, such studies have largely remained beyond the clinical public domain, mainly due to the lack of novel markers.

The reliance on single parameters for clinical differentiation often results in limited effectiveness due to inherent challenges of low sensitivity or specificity. Conversely, the development of a mathematical model integrating clinical symptoms, imaging findings, and multiple tumor markers can significantly enhance diagnostic accuracy. Our predictive model employs multivariate regression analysis to combine these variables, thereby improving the sensitivity and specificity of diagnostic evaluations and supporting more precise medical decisions. Previous studies have introduced various models to refine MPE diagnosis (15, 17); however, these often involve higher costs and the need for specialized personnel. The model’s most discriminating variable was dyspnoea, which increased the likelihood of the effusion being malignant by 14-fold (**Table 3**). MPE is linked to a median survival of 3–6 months and can significantly impair quality of life due to severe dyspnoea (18). Our model included fever and X-ray/CT findings compatible with malignancy, similar to the predictive MPE model proposed by Luis Valdes et al. (19) However, there are notable differences between the two models. Valdes et al. considered chest pain as a predictor, which was not included in our model. Their model classified 87.2% of patients with MPE, while our model achieved a higher classification accuracy of 73.4%.These differences may be attributed to several factors. Firstly, the inclusion of different clinical variables in the models, such as chest pain, could lead to varying predictive performance. Secondly, the studies employed different methodologies, which may contribute to the observed differences. Valdes et al. used logistic regression analysis to estimate the probability of MPE and considered four prognostic models with different combinations of clinical-radiological and analytical variables. In contrast, our study utilized logistic regression and focused on a single model incorporating dyspnoea, fever, X-ray/CT compatible with malignancy, pCEA, sNSE, sCEA, and pLDH. Furthermore, the patient populations and sample sizes of the two studies may have differed, potentially influencing the results. Valdes et al. included 491 pleural exudates, while our study analyzed 428 PEs. The proportions of various etiologies, such as tuberculous, malignant, and parapneumonic effusions, may have also varied between the two studies, affecting the models’ performance. Despite these differences, both studies highlight the importance of combining clinical, radiological, and analytical criteria for the accurate diagnosis of MPE. While the model proposed by Valdes et al. demonstrated good diagnostic yield, our model’s higher classification accuracy suggests that the inclusion of dyspnoea may further improve the predictive performance for MPE diagnosis.

Clinical diagnostic efficacy has been reported for pCEA, sCEA, sNSE, and pLDH in diagnosing MPE (20–22). However, LASSO regression analysis excluded SCCA levels, considered a superior method for predictor selection (23). This exclusion might reflect the lower prevalence of lung squamous cell carcinoma in this study compared to previous research (24). pCEA and sCEA are strongly associated with lung adenocarcinoma (25, 26), with pCEA being a particularly valuable marker for detecting MPE related to lung cancer (27). LDH and sNSE have also shown significant associations with lymphoma-related and small-cell lung carcinoma MPEs, respectively, offering reasonable sensitivity and specificity (28, 29).

Recent research has highlighted the significant role of metal ions in the pathogenesis and progression of various diseases. Ferroptosis and cuproptosis, particularly in the context of oncology, have been extensively studied. Innovatively, our study utilizes serum metal ion levels as biomarkers to differentiate between MPE and BPE. This suggests that metal ions may participate in the development of MPE through as yet unknown mechanisms. Consequently, further research is needed to elucidate the mechanisms by which metal ions contribute to the formation of MPE.

This investigation combined metal ions and clinical data to establish a comprehensive nomogram aimed at predicting the occurrence of MPE in patients diagnosed with PE. The nomogram incorporated seven pivotal variables: dyspnoea, fever, X-ray/CT findings indicative of malignancy, pCEA, sCEA, sNSE, and pLDH. Demonstrating substantial discriminative capability, calibration, and clinical applicability, the model provides a robust framework for distinguishing between benign and malignant pleural conditions. MPE is recognized as a frequent and serious complication associated with various malignancies, including lymphoma, and cancers of the lung, ovary, breast, and stomach. Jung et al. (30) highlighted the importance of swift and accurate discrimination between benign and malignant pleural effusions, as MPE is associated with higher morbidity and mortality rates in advanced tumors. Accurate diagnosis plays a pivotal role in guiding the selection of the most appropriate treatment strategies. For example, timely and appropriate treatment can potentially cure BPE, such as TPE and parapneumonic effusions. In contrast, malignant lung carcinoma without pleural infiltration can be effectively treated with surgery, significantly improving survival outcomes.

This study, while promising, is limited by its single-center, retrospective design and the relatively small patient cohort. The findings necessitate further validation through larger, multicentric studies to ensure the model’s applicability across diverse populations. Nonetheless, the development of this accessible and reliable predictive model represents a significant advancement in the clinical management of PE, potentially enhancing the accuracy of MPE diagnosis and informing appropriate treatment strategies.

## 5 Conclusion

Our manuscript provides a user-friendly and reliable diagnostic tool for the identification of MPE. Furthermore, our study identified significant alterations in serum calcium, copper, and zinc levels between MPE and BPE patients, highlighting the potential utility of serum metal ions as diagnostic biomarkers in MPE. These findings will enhance the precision of MPE diagnosis and facilitating personalized treatment strategies.

## Data availability statement

The original contributions presented in the study are included in the article/**Supplementary Material**. Further inquiries can be directed to the corresponding author/s.

## Ethics statement

The studies involving humans were approved by Ethics Committee of Huaian No.1 People’s Hospital, affiliated with Nanjing Medical University. The studies were conducted in accordance with the local legislation and institutional requirements. The participants provided their written informed consent to participate in this study.

## Author contributions

JJ: Data curation, Formal analysis, Investigation, Methodology, Project administration, Writing – original draft. TS: Data curation, Formal analysis, Methodology, Writing – original draft. LY: Formal analysis, Software, Visualization, Writing – original draft. KW: Software, Visualization, Writing – original draft. KJ: Software, Visualization, Writing – original draft. YJ: Investigation, Resources, Writing – original draft. SP: Investigation, Resources, Writing – original draft. YY: Writing – review & editing, Conceptualization, Resources, Supervision, Validation. CL: Conceptualization, Investigation, Project administration, Resources, Supervision, Validation, Writing – review & editing.
